# Race and Socioeconomic Status are Predictors of Survival in Patients With Metastatic Bone Disease Secondary to Breast Cancer

**DOI:** 10.1002/cnr2.70689

**Published:** 2026-10-05

**Authors:** Ashley Castan, Rosamaria Dias, Amog Mysore, David Herz, Katie Gotoff, Yazan Kadkoy, Kathleen Beebe, Joseph Benevenia, Joseph Ippolito

**Affiliations:** ^1^ Rutgers New Jersey Medical School Newark New Jersey USA

**Keywords:** breast cancer, income, metastatic bone disease, race, survival

## Abstract

**Background:**

With implementation of early detection screening methods and advances in medical treatment, there have been improvement in outcomes for patients with breast cancer. Despite this, studies have suggested potential differences in outcomes amongst various racial and ethnic groups. To our knowledge, no study has evaluated if race and demographic characteristics impact survival outcomes in patients with breast cancer metastases to the bone.

**Aims:**

We sought to answer the following questions: (1) Does race, demographic, and tumor characteristics alter survival outcomes in patients with breast metastases to the bone? (2) When stratifying by income and treatment, does race influence survival outcomes in patients with breast metastases to the bone?

**Methods and Results:**

The surveillance, epidemiology, and end results (SEER) 2016 to 2021 database was reviewed for patients with a diagnosis of breast cancer with metastases to the bone *N* = 11 833. Patients with known primary breast cancer, known hormone receptor status, known metastasis to bone, known treatment regiment, and no additional cancer diagnoses were included. Using variables within the database, this study collected information on demographics and tumor characteristics. Multivariate analyses were conducted to determine associations between race and other demographic, clinical, and prognostic factors. Kaplan–Meier survival curves were utilized to evaluate outcome by race. Annual household income below $75 000 (hazard ratio 1.111 [95% confidence interval (CI): 1.047–1.178, *p* < 0.001]), age over 65 years old (hazard ratio 1.1150 [95% CI: 1.084–1.220, *p* < 0.001]), and race (black) (hazard ratio 1.256 [95% CI: 1.161–1.359, *p* < 0.001]) were independent risk factors for decreased survival likelihood. Surgery (hazard ratio 0.554 [95% CI: 0.507–0.606, *p* < 0.001]), chemotherapy (hazard ratio 0.421 [95% CI: 0.397–0.446, *p* < 0.001]), and radiation (hazard ratio 0.940 [95% CI: 0.885–0.998, *p* < 0.05]), were independent factors that increased survival. At 12‐month follow‐up (hazard ratio 1.211 [95% CI: 1.091–1.345, *p* < 0.001]) and 5‐year follow‐up (hazard ratio 1.256 [95% CI: 1.161–1.359, *p* < 0.001]) black women had decreased survival likelihood compared to all other races. When patients were stratified by annual household income greater than $75 000, regardless of treatment, black women had decreased survival likelihood in patients younger then 65 years old (hazard ratio 1.242 [95% CI: 1.117–1.381, *p* < 0.001]) and 65 years and older (hazard ratio 1.318 [95% CI: 1.121–1.550, *p* = 0.001]). Increasing age, race, and lower annual household income were significantly associated with higher mortality. When controlling for higher income and treatment variables, black women had significantly higher mortality rates at 5‐year follow up.

**Conclusion:**

Despite improved treatment methods, disparities among patients with metastatic breast cancer to bone exists. Our findings demonstrate that different sub‐groups experience different mortality rates. Further research must be conducted to understand the interplay between these socioeconomic variables and survival likelihood in metastatic breast cancer.

## 1 Introduction

Breast cancer survival rates have improved with the implementation of early detection programs and modern treatments [1]. While the use of hormone‐targeted therapies has improved mortality rates, metastatic bone disease still imparts considerable morbidity and mortality due to various factors such as pathological fractures and spinal cord compression [2, 3, 4, 5, 6, 7, 8]. Women with localized breast cancer have reported 5‐year survival rates as high as 99%, whereas women with metastatic disease have a 5‐year survival rate of about 31% [9]. Mortality differences have been shown in women with breast cancer between various racial and socioeconomic status (SES) groups, regardless of biologic similarities, but few have studied the interaction of both SES and ethnicity [10, 11, 12, 13, 14].

While studies have shown that being in a lower SES group is associated with worse health outcomes, there remains a gap in knowledge as to why Black women have not made the same progress in survival improvement as other racial groups [15, 16, 17]. Considerable emphasis has been placed on improving access to care for all, though countries with universal healthcare show similar disparities. In Canada, for example, despite nationalized coverage, racial minority and economically disadvantaged patients continue to experience meaningfully worse access to and outcomes from breast cancer care [18]. Clear mortality differences have been established in women with breast cancer, but studies are limited on if this difference translates to women with breast cancer and bone metastasis. To our knowledge, no study has evaluated if race and demographic characteristics impact survival outcomes in patients with metastatic breast cancer to the bone. We sought to answer the following questions: (1) Does race, patient demographics, and tumor characteristics alter survival outcomes in patients with metastatic breast cancer to the bone? (2) When controlling for income disparity, does race influence survival outcomes in patients with breast metastases to the bone?

## 2 Materials and Methods

### 2.1 Study Design and Study Population

Institutional Review Board approval was not required for this study due to utilization of a publicly available database with deidentified patient characteristics. We used the Surveillance, Epidemiology, and End Results (SEER) database. The SEER program is a population‐based cancer registry of approximately 48% of the United States population that collects data on patient demographics, primary tumor site, tumor morphology and stage at diagnosis, first course of treatment, and follow‐up [19].

### 2.2 Participants

Within SEER we began a “Survival Session” and were then prompted to choose from several subsets of database. We chose “Incidence—SEER Research Data, 17 Registries, Nov 2023 Sub (2000–2021)” because it included the most recent year compared to the other options. Statistics parameters chosen included “cause‐specific survival” with the definition of cause of death being “dead due to cancer using SEER cause‐specific death classification.” Our selection parameters included to select only: microscopically confirmed, malignant behavior, and known age and to exclude: all death certificate only and autopsy only, dead due to other causes with no survival time, age values not found in table, invalid year, and values not found for other variables in table. Case selection inclusion criteria were as follows, sex: female, age: 40–85+, race/origin: non‐Hispanic White (NHW), non‐Hispanic Black (NHB), non‐Hispanic American Indian/Alaskan native (NHAIAN), non‐Hispanic Asian or pacific islander (NHAPI), Hispanic (all races), non‐Hispanic unknown race, year of diagnosis: 2016–2021, known annual household income (AHI), known estrogen receptor (ER) status, known progesterone receptor (PR) status, known human epidermal growth factor receptor 2 (HER2) status, established metastasis to bone at diagnosis, primary site: C50.0 nipple, C50.1 central portion of breast, C50.2 upper‐inner quadrant of breast, C50.3 lower‐inner quadrant of breast, C50.4 upper‐outer quadrant of breast, C50.5 lower‐outer quadrant of breast, C50.6 axillary tail of breast, C50.8 overlapping lesion of breast, C50.9 breast not otherwise specified, known surgical history of the primary tumor, known chemotherapy history, and known radiation history (Table 1). Of the 12 065 patients that were identified using SEER*Stat, 232 were excluded due to unknown race or male sex, yielding a final cohort of 11 833 women. Excluded patients did not differ from included patients with respect to age and year of diagnosis, the only variables available for comparison given SEER's data structure (Figure 1).

**TABLE 1:** Inclusion and exclusion criteria.

	Inclusion criteria	Exclusion criteria
Population	– Female, age: 40–85+, race/origin: non‐Hispanic white (NHW), non‐Hispanic black (NHB), non‐Hispanic American Indian/Alaskan native (NHAIAN), non‐Hispanic Asian or pacific islander (NHAPI), Hispanic (all races), non‐Hispanic unknown race – Known annual household income (AHI) – Known treatment history (± surgery, ± chemotherapy, ± radiation)	– Age values not found in table
Pathology	– Microscopically confirmed, malignant behavior – Known primary breast cancer (with primary site) – Known hormone receptor status, known metastasis to the bone	– All death certificate only and autopsy only – Additional cancer diagnoses
Statistics parameters	– “Cause‐specific survival” – “Dead due to cancer using SEER cause‐specific death classification” – Year of diagnosis: 2016–2021	– Dead due to other causes with no survival time – Invalid year

**FIGURE 1:**
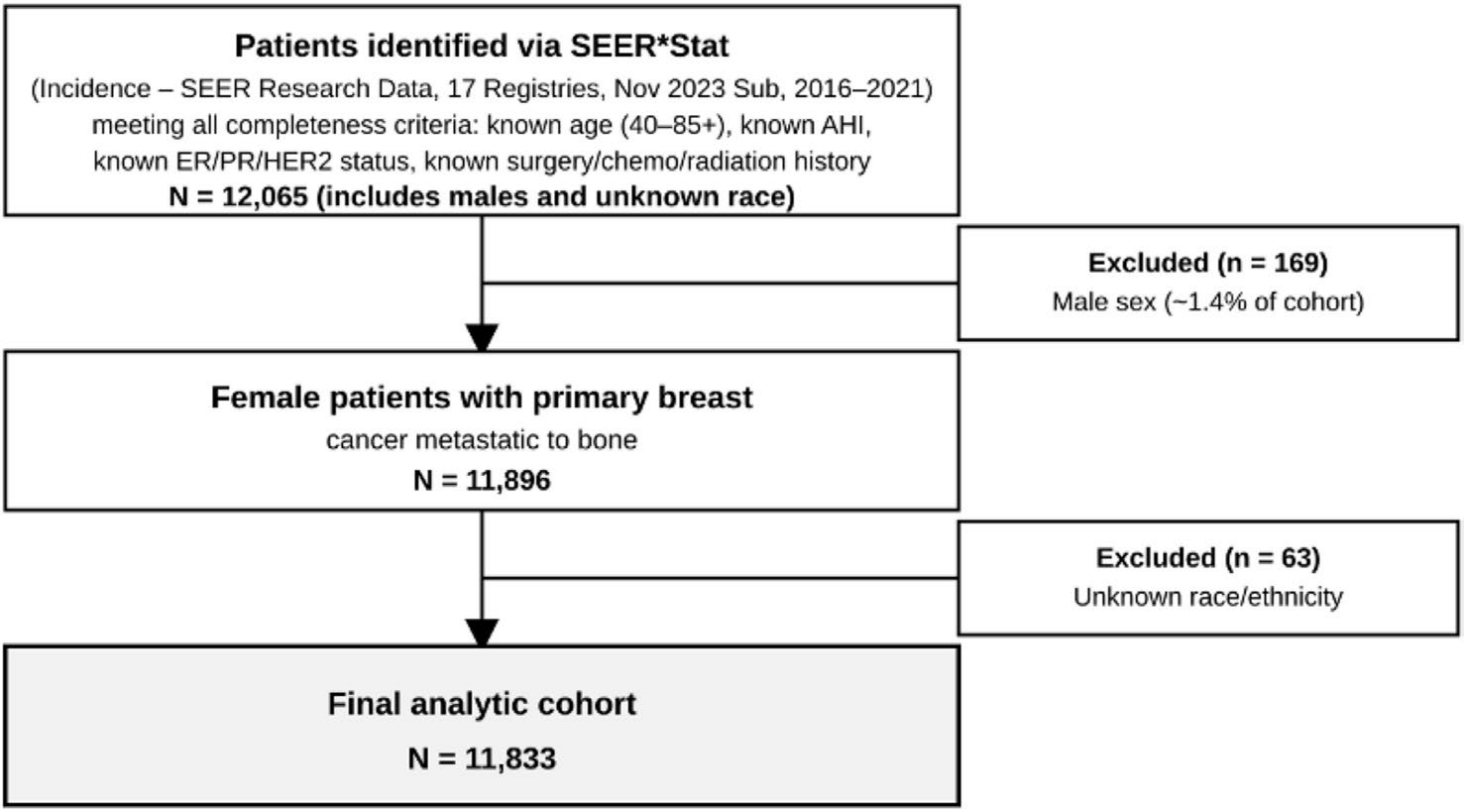
Flow diagram illustrating selection of the study cohort from the SEER database (Incidence—SEER Research Data, 17 Registries, November 2023 Sub, 2016–2021).

Men were excluded due to only making up 1.4% of the cohort to avoid confounding the results, since males with breast cancer typically have a worse prognosis regardless of stage [20]. Age selection was restricted to 40–85+ because younger women are more likely to have a genetic component which, while we did not strictly elucidate in our cohort, may have confounded results [21].

### 2.3 Statistical Analyses

Data was extrapolated from SEER as case listings and transferred into IBM SPSS software platform. Patient characteristics and demographics were summarized categorically. Age was categorized as “Under 65 years old” and “65+ years” and income as “Under $75 000” and “$75 000+.” Surgical history status was categorized as “underwent surgery” and “did not undergo surgery.” Chemotherapy history was categorized as “received chemotherapy” and “did not receive chemotherapy.” Radiation history was categorized as “received radiation” and “did not receive radiation.” Hormone receptor status was categorized by the four main subtypes, based on the combined status of progesterone, estrogen, and derived HER2 based on established molecular cancer subtype classifications [22]. ER‐negative, PR‐negative, and HER2‐positive were categorized as “HER2‐Enriched,” and ER‐negative, PR‐negative, and HER2‐negative were categorized as “Triple‐Negative.” The remaining combined status, including ER‐negative, PR‐positive, HER2‐negative, ER‐negative, PR‐positive, HER2‐positive, and ER‐positive, PR‐negative, HER2‐positive were left as the categorical names for the combined status variable. Not all patients experience the event, death, within the observation period. This is referred to as censoring and is coded the same as not experiencing the event when the patient does not experience the event by the end of the study, the individual is lost to follow‐up, or the individual withdraws from the study [23].

Disease specific survival was analyzed using the Kaplan–Meier method and the difference between survival curves was evaluated using the log‐rank test, stratifying for age and income. To determine the risk of death due to primary breast cancer with bone metastasis, Fisher's exact test and Pearson chi‐square analysis were used as indicated to compare race score against demographic and clinical/prognostic data in a univariate regression model. To evaluate the independent impact of race on survival, a multivariate Cox regression model was employed, evaluating differential survival between race populations while controlling for statistically significant demographic data, including age, income, race, molecular subtypes, and primary tumor site at 6, 12 months, and 5 years. Variables were retained in the final multivariate model based on statistically significant association with race on univariate analysis, as described above, and were additionally consistent with established prognostic factors for breast cancer survival reported in the existing literature. Given the large number of observed events in this cohort (*n* = 5019 deaths) relative to the small number of covariates included, the ratio of events to model parameters substantially exceeded conventional thresholds for avoiding overfitting. Cox proportional hazard regression models were used to obtain hazard ratios (HRs) and 95% confidence intervals (CIs) for disease‐specific survival. The significance level was set at *p* < 0.05 in all statistical tests. The proportional hazards assumption was assessed for each covariate using log‐minus‐log survival plots and time‐dependent covariate terms.

## 3 Results

### 3.1 Relationship Between Survival and Patient Characteristics

#### 3.1.1 Factors Associated With Survival

A total of 11 833 patients with primary breast cancer with metastatic bone disease were included in this analysis. Variables included in the analysis were age, annual household income (AHI), race, surgical resection history, history of chemotherapy, history of radiation, receptor status, and site of primary breast lesion (Table 2). With the numbers available, AHI below $75 000 (hazard ratio 0.900 [95% confidence interval (CI): 0.849–0.955, *p < 0.001*]), age over 65 years old (hazard ratio 1.150 [95% CI: 1.084–1.220, *p < 0.001*]), and being Black (hazard ratio 1.241 [95% CI: 1.145–1.345, *p < 0.001*]) were independent risk factors for decreased survival likelihood (Table 3). History of surgical resection (hazard ratio 0.554 [95% CI: 0.507–0.606, *p < 0.001*]), chemotherapy (hazard ratio 0.421 [95% CI: 0.397–0.446, *p < 0.001*]), and radiation (hazard ratio 0.940 [95% CI: 0.885–0.998, *p < 0.05*]) were all individually associated with increased survival.

**TABLE 2:** Demo‐clinical characteristics predictive for disease specific survival of patients with breast cancer metastasizing to the bone.

		Race cohort						*p*
		Non‐Hispanic White	Non‐Hispanic Black	Hispanic (all races)	Non‐Hispanic Asian or Pacific Islander	Non‐Hispanic American Indian/Alaska Native	Total	
Age cohorts in years *N* (%)	Under 65 years old	3498 (29.6)	988 (8.3)	962 (8.1)	689 (5.8)	51 (0.4)	6188 (52.3)	**< 0.001**
	65+ years	4116 (34.8)	639 (5.4)	525 (4.4)	337 (2.8)	28 (0.2)	5645 (47.7)	
	Total	7614	1627	1487	1026	79	11 833	
Income cohorts in $*N* (%)	Under $75 000	2642 (22.3)	771 (6.5)	429 (3.6)	119 (1.0)	35 (0.3)	3996 (33.8)	**< 0.001**
	$75 000+	4972 (42.0)	856 (7.2)	1058 (8.9)	907 (7.7)	44 (0.4)	7837 (66.2)	
	Total	7614	1627	1487	1026	79	11 833	
Primary tumor site, *N* (%)	Nipple	25 (0.2)	11 (0.9)	11 (0.9)	1 (0)	0 (0)	48 (0.4)	**< 0.001**
	Central portion	497 (4.2)	91 (0.8)	89 (0.8)	61 (0.5)	10 (0.1)	748 (6.3)	
	Upper‐inner quadrant	451 (3.8)	104 (0.9)	100 (0.8)	68 (0.6)	6 (0)	729 (6.2)	
	Lower‐inner quadrant	273 (2.3)	52 (0.4)	40 (0.3)	25 (0.2)	3 (0)	392 (3.3)	
	Upper‐outer quadrant	1788 (15.1)	401 (3.4)	337 (2.8)	207 (1.7)	22 (1.9)	2755 (23.3)	
	Lower‐outer quadrant	416 (3.5)	65 (0.5)	76 (0.6)	48 (0.4)	4 (0)	609 (5.1)	
	Axillary tail	78 (0.7)	14 (0.1)	2 (0)	5 (0)	0 (0)	99 (0.8)	
	Overlapping lesion	1531 (12.9)	318 (2.7)	311 (2.6)	207 (1.7)	11 (0.1)	2378 (20.1)	
	Not otherwise specified	2555 (21.6)	571 (4.8)	521 (4.4)	404 (3.4)	23 (0.2)	4074 (34.4)	
	Total	7614	1627	1487	1026	79	11 833	
Molecular subtype, *N* (%)	ER+PR+HER2+	704 (5.9)	163 (1.4)	143 (1.2)	101 (0.9)	9 (0.1)	1120 (9.5)	**< 0.001**
	ER+PR+HER2−	4555 (38.5)	782 (6.6)	780 (6.7)	543 (4.6)	45 (0.4)	6705 (56.7)	
	ER−PR+HER2+	29 (0.2)	6 (0)	3 (0)	11 (0.1)	0 (0)	49 (0.4)	
	ER−PR+HER2−	31 (0.2)	12 (0.1)	10 (0.1)	2 (0)	0 (0)	55 (0.5)	
	ER+PR−HER2+	350 (3.0)	85 (0.7)	87 (0.7)	58 (0.5)	3 (0)	583 (4.9)	
	ER+PR−HER2−	905 (7.6)	214 (1.8)	186 (1.6)	120 (1.0)	11 (0.1)	1436 (12.1)	
	ER−PR−HER2+	412 (3.5)	113 (1.0)	136 (1.1)	94 (0.8)	3 (0)	758 (6.4)	
	ER−PR−HER2−	628 (5.3)	252 (2.1)	142 (1.2)	97 (0.8)	8 (0.1)	1126 (9.5)	
	Total	7614	1627	1487	1026	79	11 833	
Surgery, *N* (%)	Received surgery	1182 (10.2)	233 (2.0)	252 (2.2)	159 (1.4)	13 (0.1)	1839 (15.9)	**< 0.001**
	Did not receive surgery	6281 (54.1)	1362 (11.7)	1210 (10.4)	844 (7.3)	64 (0.6)	9761 (84.1)	
	Total	7463	1595	1462	1003	77	11 600	
Chemotherapy, *N* (%)	Received chemotherapy	4904 (41.4)	1069 (9.0)	1009 (8.5)	719 (6.1)	50 (0.4)	7751 (65.5)	**< 0.001**
	Did not receive chemotherapy	2710 (22.9)	558 (4.7)	478 (4.0)	307 (2.6)	29 (0.2)	4082 (34.5)	
	Total	7614	1627	1487	1026	79	11 833	
Radiation, *N* (%)	Received radiation	2530 (21.7)	499 (4.3)	514 (4.4)	362 (3.1)	32 (0.3)	3937 (33.8)	**< 0.001**
	Did not receive radiation	4969 (42.6)	1105 (9.5)	949 (8.1)	645 (5.5)	47 (0.4)	7715 (66.2)	
	Total	7499	1604	1463	1007	79	11 652	

*Note:* Bold values indicate the statistically significant.

**TABLE 3:** Multivariate cox regression analysis of 5‐year overall survival of patients with primary breast cancer presenting with metastatic bone disease.

	Hazard ratio	95 confidence interval	Significance
Race			
Non‐Hispanic Black	1.241	1.145–1.345	< 0.001
Hispanic (all races)	0.958	0.913–1.090	0.958
Non‐Hispanic Asian or Pacific Islander	0.865	0.774–0.967	0.011
Non‐Hispanic American Indian/Alaska Native	1.097	0.778–1.547	0.597
Non‐Hispanic White	^*^	^*^	^*^
Primary tumor site			
Lower‐outer quadrant	0.831	0.716–0.963	0.014
Upper‐inner quadrant	0.939	0.820–1.074	0.358
Lower‐inner quadrant	0.940	0.794–1.115	0.479
Nipple	1.232	0.773–1.964	0.380
Central portion	1.019	0.898–1.156	0.769
Axillary	1.000	0.720–1.389	0.999
Overlapping	1.029	0.942–1.124	0.523
Not otherwise specified	1.287	1.194–1.387	< 0.001
Upper‐outer quadrant	^*^	^*^	^*^
Age			
Under/over 65 years old	1.150	1.084–1.220	< 0.001
Income			
Under/over 75 K	0.900	0.849–0.955	< 0.001
Molecular subtype			
ER+PR+HER2+	1.069	0.961–1.188	0.219
ER−PR+HER2+	1.934	1.313–2.849	< 0.001
ER−PR+HER2−	4.880	3.552–6.706	< 0.001
ER+PR−HER2+	1.301	1.136–1.490	< 0.001
ER+PR−HER2−	1.588	1.459–1.730	< 0.001
ER−PR−HER2+	2.035	1.815–2.282	< 0.001
ER−PR−HER2−	4.159	3.819–4.53	< 0.001
PR+ER+HER2−	^*^	^*^	^*^
Surgery			
Did not receive/received	0.554	0.507–0.606	< 0.001
Chemotherapy			
Did not receive/received	0.421	0.397–0.446	< 0.001
Radiation			
Did not receive/received	0.940	0.885–0.998	< 0.05

^*^ Indicates that non‐hispanic white women was the reference group.

#### 3.1.2 Relationship Between Survival and Race

Overall patient survival was analyzed at 6‐month follow‐up, 12‐month follow‐up, and 5‐year follow‐up. At 6‐month follow‐up, Hispanic (hazard ratio 0.856 [95% CI: 0.734–0.999, *p < 0.05*]) and Asian (hazard ratio 0.723 [95% CI: 0.590–0.887, *p = 0.002*]) women had increased survival regardless of income and treatment compared to White women. Black women compared to Hispanic women had increased risk of death (hazard ratio 1.251 [95% CI: 1.041–1.541, *p < 0.05*]). Black women had overall decreased survival compared to all other races women at 12‐month (hazard ratio 1.211 [95% CI: 1.091–1.345, *p = 0.004*]) and 5‐year follow‐up (hazard ratio 1.256 [95% CI: 1.161–1.359, *p < 0.001*]) (Figure 2).

**FIGURE 2:**
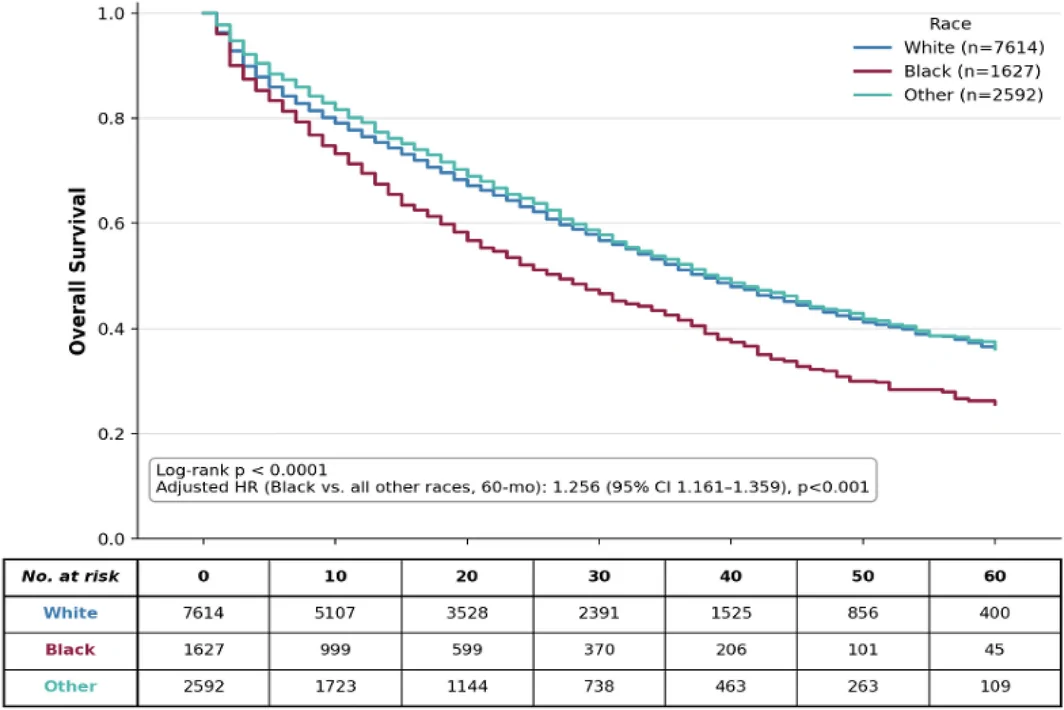
Kaplan–Meier curve comparing disease‐specific survival among non‐Hispanic Black women versus non‐Hispanic White women and all other races combined, in the full cohort of patients with primary breast cancer metastatic to bone (*N* = 11 833). Black women had significantly lower survival probability throughout follow‐up (5‐year HR 1.256, 95% CI: 1.161–1.359, *p* < 0.001; log‐rank *p* < 0.0001).

#### 3.1.3 Relationship Between Survival, Annual Household Income, and Age

At 6‐month follow‐up, an AHI of less than $75 000 (hazard ratio 1.204 [95% CI: 1.038–1.397, *p* < 0.001]) and being 65 years and older (hazard ratio 1.120 [95% CI: 1.013–1.239, *p* < 0.05]) were found to be associated with increased mortality for all women. At 12‐month follow‐up, there was similar findings overall for an AHI of less than $75 000 (hazard ratio 1.152 [95% CI: 1.063–1.250, *p* < 0.001]) and being 65 years and older (hazard ratio 1.137 [95% CI: 1.047–1.234, *p* = 0.002]). At 5‐year follow‐up, there was similar findings overall for an AHI of less than $75 000 (hazard ratio 1.111 [95% CI: 1.047–1.178, *p* < 0.001]) and being 65 years and older (hazard ratio 1.150 [95% CI: 1.084–1.220, *p* = 0.002]).

At 5‐years follow‐up, when controlling for treatment and stratifying by AHI of $75 000 or greater, Black women had an increased risk of mortality when compared to all other races (hazard ratio 1.256 [95% CI: 1.161–1.359, *p* < 0.001]). This remained true when further stratifying this AHI group by age of less than 65 years old (hazard ratio 1.242 [95% CI: 1.117–1.381, *p* < 0.001]) and 65 years and older (hazard ratio 1.318 [95% CI: 1.121–1.550, *p* < 0.001]) (Figures 3 and 4).

**FIGURE 3:**
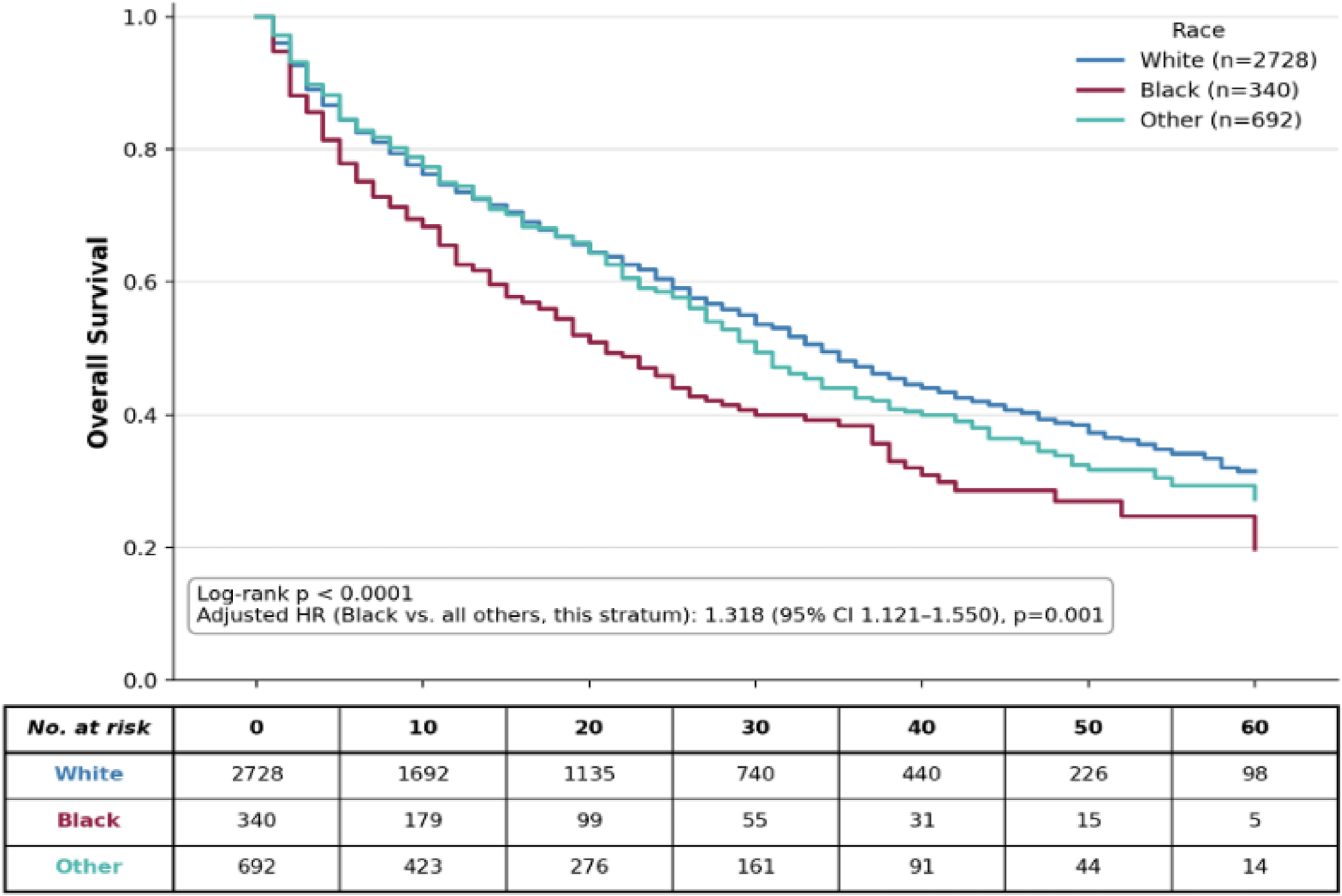
The Kaplan–Meier curve shows the decreased survival of Black women 65 years and older years old with an annual household income of $75 000 and over compared to White women and all other races also 65 years and older with the same annual household income grouping (HR 1.318, 95% CI: 1.121–1.550, *p* < 0.001; log‐rank *p* < 0.0001).

**FIGURE 4:**
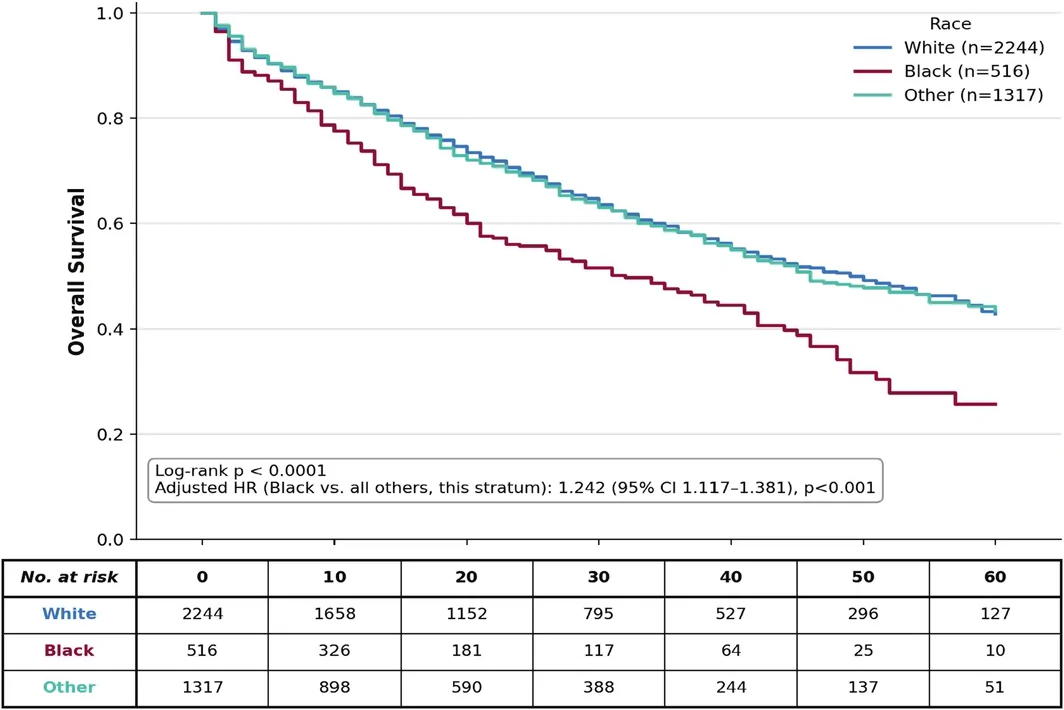
The Kaplan Meier curve shows the decreased survival of Black women less than 65 years old with an annual household income of $75 000 and over compared to White women and all other races also under 65 years old with the same annual household income grouping (HR 1.242, 95% CI: 1.117–1.381, *p* < 0.001; log rank *p* < 0.0001).

At 5‐years follow‐up, when controlling for treatment and stratifying by AHI of less than $75 000, Black women had an increased risk of mortality (hazard ratio 1.184 [95% CI: 1.057–1.327, *p* = 0.004]). This remained true when further stratifying this AHI group by age of less than 65 years old (hazard ratio 1.175 [95% CI: 1.008–1.369, *p* < 0.05]). When stratifying by age 65 years and older, Black women compared to Hispanic women (hazard ratio 1.484 [95% CI: 1.102–2.000, *p* = 0.009]) had an increased mortality risk (Table 4).

**TABLE 4:** Multivariate cox regression analysis of 6‐month, 12‐month, and 60‐month overall survival of patients with primary breast cancer presenting with metastatic bone disease.

	Hazard ratio	95 confidence interval	Significance
6‐month overall survival			
Non‐Hispanic Black versus all other races	1.130	0.994–1.286	0.063
Hispanic (all races) versus all other races	0.870	0.748–1.011	0.069
Non‐Hispanic Black versus Hispanic (all races)	1.251	1.041–1.504	0.017
Non‐Hispanic White versus all other races	1.088	0.983–1.204	0.105
AHI ≥ $75 000 versus AHI < $75 000 all races	0.831	0.716–0.963	0.014
Age ≥ 65 years old versus < 65 years all races	1.120	1.013–1.239	0.027
Surgery all races	0.290	0.231–0.365	< 0.001
Chemotherapy all races	0.187	0.168–0.208	< 0.001
Radiation all races	0.793	0.713–0.882	< 0.001
12‐month overall survival			
Non‐Hispanic Black versus all other races	1.211	1.091–1.345	< 0.001
Hispanic (all races) versus all other races	0.907	0.803–1.025	0.117
Non‐Hispanic Black versus Hispanic (all races)	1.277	1.101–1.482	0.001
Non‐Hispanic White versus all other races	1.009	0.929–1.096	0.837
AHI ≥ $75 000 versus AHI < $75 000 all races	0.868	0.800–0.941	< 0.001
Age ≥ 65 years old versus < 65 years old all races	1.137	1.047–1.234	0.002
Surgery all races	0.391	0.355–0.457	< 0.001
Chemotherapy all races	0.262	0.241–0.285	< 0.001
Radiation all races	0.865	0.794–0.942	< 0.001
60‐month overall survival			
Non‐Hispanic Black versus all other races	1.256	1.161–1.359	< 0.001
Hispanic (all races) versus all other races	0.975	0.894–1.063	0.564
Non‐Hispanic Black versus Hispanic (all races)	1.241	1.114–1.382	< 0.001
Non‐Hispanic White versus all other races	0.945	0.890–1.003	0.064
AHI ≥ $75 000 versus AHI < $75 000 all races	0.900	0.849–0.955	< 0.001
Age ≥ 65 years old versus < 65 years old all races	1.150	1.084–1.220	< 0.001
Surgery all races	0.554	0.507–0.606	< 0.001
Chemotherapy all races	0.421	0.397–0.446	< 0.001
Radiation all races	0.940	0.885–0.998	0.044

#### 3.1.4 Relationship Between Survival and Treatment

At 6‐months, overall history of surgical resection (hazard ratio 0.290 [95% CI: 0.231–0.365, *p < 0.001*]), history of chemotherapy (hazard ratio 0.187 [95% CI: 0.168–0.208, *p < 0.001*]), and history of radiation (hazard ratio 0.793 [95% CI: 0.713–0.882, *p < 0.001*]) were each associated with increased survival. At 12‐month follow‐up, the same trend was seen: history of surgical resection (hazard ratio 0.391 [95% CI: 0.335–0.457, *p < 0.001*]), history of chemotherapy (hazard ratio 0.262 [95% CI: 0.241–0.285, *p < 0.001*]), and history of radiation (hazard ratio 0.865 [95% CI: 0.794–0.942, *p < 0.001*]). At 5‐year follow‐up, the same associations were observed: history of surgical resection (hazard ratio 0.554 [95% CI: 0.507–0.606, *p < 0.001*]), history of chemotherapy (hazard ratio 0.421 [95% CI: 0.397–0.446, *p < 0.001*]), and history of radiation (hazard ratio 0.940 [95% CI: 0.885–0.998, *p < 0.05]*).

#### 3.1.5 Relationship Between Survival and Hormone Receptor Status

Mortality risk calculations performed for all possible hormone receptor statuses (Table 3). The hormone statuses with the highest risk for mortality were ER−PR+HER2− (hazard ratio 4.880 [95% CI: 3.552–6.706, *p* < 0.001]) and ER−PR−HER2− (hazard ratio 4.159 [95% CI: 3.819–4.530, *p* < 0.001]).

Non‐Hispanic Black women had approximately twice the odds of attaining triple negative (ER−PR−HER2−) molecular subtype compared to White women (OR 2.04, 95% CI: 1.74–2.39). A similar, though not statistically significant, trend was observed for ER−PR+HER2− subtype (OR 1.82, 95% CI: 0.93–3.55).

#### 3.1.6 Relationship Between Survival and Primary Tumor Site

Mortality risk calculations were done based on primary tumor site and are reflected in Table 3. We did not stratify site by race or AHI. When the primary site was the lower outer quadrant (hazard ratio 0.831 [95% CI: 0.716–0.963, *p < 0.05*]), mortality risk was decreased.

#### 3.1.7 Proportional Hazard Assumption Testing

The proportional hazards assumption was tested for each covariate that was included in the multivariate model. No evidence of a violation was observed for race, the primary variable of interest (*p* = 0.330); time‐dependent effects were identified for age, income, surgery, chemotherapy, and radiation (*p* < 0.001).

## 4 Discussion

There remains disparity in overall breast cancer survival outcomes in Black women [2, 3]. A study by Hill et al. reported that the 5‐year survival of Black women has lagged behind their white counterparts by up to 20 years [18, 24]. In a paper by Giaquinto et al., this was attributed to limited access by Black women and the need for improved outreach and Medicare reform [3]. It is known from literature that Black women are more likely to present at later stages of disease [25]. We found that even when controlling for treatment, Black race remained independently associated with worse disease‐specific survival compared to White women and all other races combined, independent of the treatment variables captured in the database. The disparity observed in this study should be understood within the broader context of structural determinants of health rather than as an inherent effect of race itself. Structural racism, manifested through residential segregation, unequal distribution of high‐quality cancer care facilities, and disparities in insurance coverage, has been identified as a major contributor to racial gaps in breast cancer outcomes, independent of clinical factors [26]. It is worth noting that race is increasingly recognized in the previous oncology literature as a social construct or proxy for those unmeasured structural and socioeconomic exposures rather than a biological determinant of the prognosis or outcome itself [27]. Our results also demonstrate that at follow‐up, Hispanic women with metastatic bone disease have overall decreased mortality compared to Black women, which was consistent with existing literature [2].

Hormone receptor status was found to impact survival. Being PR+ alone was seen to have the highest impact on mortality, followed by being triple negative. Studies have shown that being positive for only one non estrogen hormone receptor has been associated with decreased survival and increased recurrence [28]. Triple negative breast cancer, historically, has been associated with poor outcomes and survival due to the limited therapeutic options [29]. Most existing literature on the impact of hormone receptor status and survival does not specifically look at patients with metastatic bone disease, which itself is associated with increased mortality.

Molecular subtype distribution may partially explain the survival disparity observed in this cohort. In our data, Black women had approximately twice the odds of a triple‐negative (ER−PR−HER2−) breast cancer compared to White women (OR 2.04, 95% CI: 1.74–2.39), consistent with a substantial body of literature describing a higher prevalence of triple‐negative and basal‐like breast cancer among Black women [30]. The disparity has itself been linked to structural determinants: Black women residing in US states with greater structural racism, measured across domains such as education and disenfranchisement, have been shown to have higher odds of a triple‐negative diagnosis than Black women in states with lower structural racism, an association that is not observed to the same degree amongst other races [31]. Neighborhood‐level socioeconomic and racial segregation measure have similarly been linked to triple‐negative breast cancer risks [32]. However, race remained an independent predictor of mortality even after adjusting for molecular subtype in our multivariate model, suggesting that subtype distribution alone does not fully account for the survival disparity observed in this cohort.

Additionally, prior studies have demonstrated that socioeconomic status directly correlates with health outcomes and access to care [13]. However, our data suggests that SES alone does not explain worse outcomes seen among Black women. Black women who had an annual household income of greater than $75 000, regardless of age and treatment, had increased risk of death compared to all other races. Previously, differences in outcomes have been largely attributed to lack of access, with higher income being associated with decreased death from breast cancer and increased mammogram screenings [33, 34, 35, 36]. However, the difference in mortality risk was seen even when controlling for treatment between the races. Our findings suggest that the gap that exists in outcomes of Black women with metastatic bone lesions cannot be entirely attributed to SES or stage of diagnosis.

Significant strides have been made to improve the outcomes of patients with primary breast cancer, but there remains a gap in the outcomes of Black women with metastatic bone disease. More research is needed to better understand the significant difference in mortality of Black women with primary breast cancer with metastatic bone lesions.

### 4.1 Limitations

This study should be considered in light of several limitations. As with any large database study, several database groupings can be chosen within SEER; while we selected the version offering the most recent data, another version may yield different results. SEER does not capture several clinically important prognostic variables, including comorbidities, performance status, extent/burden of metastatic disease, presence of visceral metastases, and details of systemic therapy (e.g., endocrine therapy, CDK 4/6 inhibitor use, HER2‐targeted agents), introducing potential residual confounding that could not be addressed in the analysis. SEER also only provides data on surgery of the primary tumor, not on metastatic sites. Additionally, our inclusion criteria required complete data on income, receptor status, and treatment variables; this may have introduced selection bias by excluding patients with missing data, who may be more likely to come from disadvantaged or underrepresented groups. Finally, because race is a social construct that may serve as a proxy for unmeasured structural and biological factors, our findings regarding race and survival should be interpreted as associative rather than causal. While the proportional hazards assumption held for race, our primary exposure of interest, formal testing identified time‐varying effects for age, income, and treatment variables (surgery, chemotherapy, and radiation). As a result, the hazards ratios reported for these covariates should be interpreted as average effects over the study period rather than as constant over time; our finding of decreased survival likelihood at multiple individual timepoints (6, 12‐month, and 5‐year follow‐up) partially address this by capturing effects at distinct points in follow‐up timeline.

## 5 Conclusions

This study found that non‐Hispanic Black race was independently associated with worse disease‐specific survival among women with metastatic breast cancer to bone, regardless of age, household income, or treatment received. These findings suggest that factors beyond late‐stage presentation and access to care, potentially including structural, biological, or unmeasured clinical factors, may contribute to the increased mortality risk observed among non‐Hispanic Black women. Future research should be conducted to explore the specific factors that contribute to this increased mortality risk.

## Author Contributions

**Ashley Castan:** conceptualization, investigation, writing – original draft, writing – review and editing, visualization, methodology, formal analysis, data curation. **Rosamaria Dias:** writing – review and editing, data curation, conceptualization, investigation. **Amog Mysore:** writing – review and editing, visualization, formal analysis, data curation. **David Herz:** conceptualization, investigation, software, data curation, formal analysis. **Katie Gotoff:** conceptualization, investigation, writing – review and editing, visualization, validation. **Yazan Kadkoy:** conceptualization, writing – review and editing, visualization, validation, supervision. **Kathleen Beebe:** conceptualization, investigation, supervision, formal analysis, writing – review and editing. **Joseph Benevenia:** conceptualization, investigation, supervision. **Joseph Ippolito:** conceptualization, investigation, writing – review and editing, formal analysis, supervision.

## Funding

The authors have nothing to report.

## Conflicts of Interest

The authors declare no conflicts of interest.

## Data Availability

The data that support the findings of this study are available from the corresponding author upon reasonable request.
